# Incorporating assessment of the cervical facet joints in the modified Stoke ankylosing spondylitis spine score is of additional value in the evaluation of spinal radiographic outcome in ankylosing spondylitis

**DOI:** 10.1186/s13075-017-1285-1

**Published:** 2017-04-26

**Authors:** Fiona Maas, Suzanne Arends, Elisabeth Brouwer, Hendrika Bootsma, Reinhard Bos, Freke R. Wink, Anneke Spoorenberg

**Affiliations:** 1 Rheumatology and Clinical Immunology, University Medical Center Groningen, University of Groningen, P.O. Box 30.001, 9700 RB Groningen, The Netherlands; 2 Rheumatology, Medical Center Leeuwarden, Leeuwarden, The Netherlands

**Keywords:** Ankylosing spondylitis, Spinal damage, Outcome measure, Radiography, OMERACT

## Abstract

**Background:**

To aim was to investigate the additional value of incorporating the de Vlam cervical facet joint score in the modified ankylosing spondylitis (AS) spine score (mSASSS) for the evaluation of spinal radiographic outcome in AS.

**Method:**

Baseline and 4-year radiographs from 98 consecutive patients from the Groningen Leeuwarden AS (GLAS) cohort, who had AS treated with TNF-α inhibitors, were scored by two readers; the vertebral bodies were assessed according to the mSASSS (0–72) and cervical facet joints (C2–C7) were assessed according to the method of de Vlam (0–15). The combined AS spine score (CASSS) was calculated as the sum of both total scores (range 0–87) and compared with the original mSASSS according to three aspects of the Outcome Measures in Rheumatology Clinical Trials (OMERACT) filter: feasibility, discrimination, and truth.

**Results:**

Feasibility: the CASSS was calculated in 91% of the patients. No additional radiographs were necessary and the assessment took only a few extra minutes. Discrimination: both scoring methods had excellent inter-observer reliability (intra-class correlation coefficient (ICC) status scores >0.99, progression scores 0.92). Incorporating the cervical facet joints did not result in an increase in measurement error. The CASSS detected more patients with definite damage (61% vs. 57%) and definite progression (55% vs. 48%). Truth: higher CASSS scores at baseline and higher progression scores were seen in 41 (46%) and 22 (25%) patients, respectively. Cervical rotation correlated better with cervical CASSS than with cervical mSASSS (Spearman’s rho = 0.68 vs. 0.59).

**Conclusions:**

The CASSS is a relevant and easy modification of the mSASSS. It captures more patients with AS who have spinal radiographic damage and progression, which is of great additional value in the evaluation of radiographic outcome in this heterogeneous and overall slowly progressing disease.

## Background

Evaluation of structural damage is essential for the diagnosis and monitoring of ankylosing spondylitis (AS). The primary locations of interest are the sacroiliac joints and the spine. Changes in the sacroiliac joints are especially important for diagnosis, whereas changes in the spine are more sensitive for monitoring disease outcome [1]. Many sites in the spine can be evaluated including the vertebral bodies, vertebral margins and ligaments, intervertebral spaces, and the facet joints [1].

Until now, the modified Stoke AS spine score (mSASSS) is found to be the best and most widely used scoring method to assess evidence of spinal damage on conventional radiographs in randomized controlled trials (RCTs) and cohort studies [2]. This scoring method includes the anterior elements of the cervical and lumbar spine. Despite good reliability of the scoring method, the mSASSS has only moderate sensitivity to change [3]. In our previous analysis in 210 patients with AS treated with TNF-α, the smallest detectable change (SDC) for 2-year progression rates was larger than the mean progression at the group level (2.3 vs. 1.6, respectively) [4]. SDC refers to the change in scores that can be detected without measurement error [5]. This indicates that the mean observed progression at the group level might be a result of measurement error instead of representing “real” progression. For progression over a time period ≥4 years, the SDC was smaller than the mean progression suggesting that at least 4 years of follow up is needed for sufficient discriminatory power of the mSASSS to detect changes.

The change in radiographic evidence of damage is an important subject of interest in monitoring the effects of treatment on spinal radiographic progression in clinical studies. However, this is hampered by the nature of AS, a slowly and very heterogeneously progressing disease [6, 7]. Therefore, researchers have attempted previously to improve the performance of the mSASSS by incorporating more spinal elements into the scoring method. Including anterior elements of the lower part of thoracic spine (T10–T12) in the mSASSS has resulted in higher status and progression scores and a larger proportion of patients identified with (new) syndesmophytes compared to the original mSASSS [8]. However, in a longitudinal study including 809 radiographs from 195 patients with AS, in most cases the three additional thoracic vertebrae were not visible on lateral radiographs of the lumbar spine (in 64% of the radiographs), which is an important limitation to feasibility [9].

Besides the anterior elements of the vertebral bodies as scored in the mSASSS, the facet joints or zygapophyseal joints are frequently affected in AS [10–12] The presence of facet joint damage is associated with longer disease duration, greater disease activity, and most importantly, with worse spinal mobility [10, 12]. Longitudinal studies on the course of spinal mobility showed that the majority of patients with AS develop moderate to severe impairment of spinal mobility. Patients with severe spinal restrictions had severe spinal deformity [13, 14]. Due to the anatomy of the facet joints, damage to these joints results in reduced cervical rotation [12]. Reduced cervical rotation hampers patients in all kinds of daily activities. Therefore, inclusion of the facet joints in a radiographic scoring method seems clinically relevant.

The presence and development of radiographic evidence of damage in the facet joints is not always concurrent with damage or radiographic progression of the anterior elements of the spine [10, 12]. In our recent study in 99 patients with AS treated with TNF-α inhibitors, we found that the majority of patients who developed new ankylosis of the cervical facet joints did not develop new syndesmophytes in the cervical spine [12]. These findings indicate that evaluation of the cervical facet joints in addition to the anterior elements of the spine could improve construct validity and possibly also the sensitivity of the mSASSS to change.

Radiographic damage of facet joints can be scored with the method of de Vlam et al. [10] In particular, the cervical facet joints are easy to score on lateral radiographs [10, 15], which is in contrast with scoring the lumbar facet joints. For this, three-quarter-oblique lumbar spine radiographs are needed. However, the reproducibility of oblique radiographic assessment might be limited [16]. Most important, the cervical facet joints can be scored more reliably than the lumbar facet joints [10].

The aim of the present study was to investigate the additional value of incorporating the de Vlam cervical facet joint score in the mSASSS for the evaluation of spinal radiographic outcome in AS. In order to investigate the applicability of a new scoring method, the Outcome Measures in Rheumatology Clinical Trials (OMERACT) filter has been proposed, which includes three aspects: feasibility, discrimination, and truth [17, 18]. The combined AS spine score (CASSS), a composite scoring method, was compared with the original mSASSS according to these three aspects of the OMERACT filter.

## Methods

The present analysis was performed in 98 consecutive patients from the Groningen Leeuwarden AS (GLAS) cohort study, who had AS and for whom lateral-projection radiographs of the cervical and lumbar spine were available at baseline and after 4 years of follow up. All patients were treated with TNF-α inhibitors because of persistent high disease activity despite conventional treatment. As described previously, the GLAS cohort is a large ongoing, prospective, longitudinal, observational, Dutch cohort study in which patients are followed according to a fixed protocol and treated according to the national and international guidelines [4, 12]. Patients were 18 years or older, and fulfilled the modified New York criteria for AS and the assessment of spondyloarthritis (ASAS) criteria for starting TNF-α blocking therapy [19].

The GLAS cohort was approved by the local ethics committees of the Medical Center Leeuwarden (MCL) and the University Medical Center Groningen (UMCG), and all patients gave written informed consent according to the Declaration of Helsinki.

### Radiological assessments

Lateral radiographs of the cervical and lumbar spine were scored independently by two trained readers (FM and RC). The anterior corners of the vertebrae (lower C2 to upper T1) were scored according to the mSASSS (range 0–72): 0 = normal, 1 = erosion, sclerosis, and/or squaring, 2 = non-bridging syndesmophyte, and 3 = bridging syndesmophyte [2, 3]. In addition, the cervical facet joints (C2–C3 to C6–C7) were scored according to the method of de Vlam et al.: 0 = normal, 1 = joint space narrowing or erosion, 2 = partial blurring or ankylosis, and 3 = complete blurring or ankylosis (range 0–15) [10, 12]. Abnormalities related to degenerative changes, defined as a reduction in intervertebral disk space height and horizontal bone spurs (>45°) at the facet joints and/or vertebral bodies [20], were not taken into account and therefore not scored. All patient identifying information and the performance dates were removed from the radiographs in order to blind readers to patient characteristics and time sequence. Scoring radiographic progression with unknown time sequence was used to diminish reader bias, as all patients were treated with TNF-α inhibitors.

For both scoring methods, a total score was calculated based on the average of the total scores of both readers. A third reader (AS) independently reassessed the radiographs in case of discrepancy in the total scores above the 95% limits of agreement or when there was discrepancy in the presence of non-bridging or bridging syndesmophytes and the presence of partial or complete ankylosis of the facet joints. The score of the primary reader closest to the third reader was used.

A composite score of the mSASSS and the total facet joint score was calculated by summing the total scores of both scoring methods. This combination score was named the combined AS spine score (CASSS) and had a scoring range of 0–87. For the mSASSS, the presence or development of definite damage was defined as the presence or development of ≥1 non-bridging or bridging syndesmophyte. In the CASSS, the presence or development of definite damage was defined as the presence or development of (partial) ankylosis in ≥1 facet joints or the presence or development of ≥1 non-bridging or bridging syndesmophytes [12].

### OMERACT filter

The OMERACT filter consists of three aspects in order to judge the applicability of measurement instruments: feasibility, discrimination, and truth [17, 18]. The CASSS was compared with the original mSASSS as the gold standard according to these three aspects of the OMERACT filter.

#### Feasibility

The feasibility aspect of the OMERACT filter focuses on the question: “Can the measure be applied easily, given constraints of time, money, and interpretability?” To answer this question, information was given about required radiographs, the time needed for scoring, and the ability to score and obtain total scores for both the mSASSS and CASSS.

#### Discrimination

The discrimination aspect of the OMERACT filter addresses the question: “Does the measure discriminate between situations of interest?” These situations can relate to states at one time point or change in states over time. The discrimination aspect captures reliability and sensitivity to change. Inter-observer reliability for the mSASSS and the CASSSS status and progression scores was analyzed using the ICC (two-way mixed effects model, single measures, absolute agreement). Bland and Altman plots were constructed and the SDCwas calculated as:

$$ 1.96\ast {\mathrm{SD}}_{\varDelta \left(\mathrm{Progression}\ \mathrm{score}\right)/\left(\surd 2\ast \surd \mathrm{k}\right)} $$

in which *k* represents the number of readings) [4], to explore systemic error and measurement error. Cohen’s kappa and the percentage of absolute agreement were used to explore the inter-observer reliability of measuring the presence or development of definite damage according to both scoring methods. ICCs and kappas of 0.00–0.20 were interpreted as poor, of 0.20–0.40 as fair, of 0.40–0.60 as moderate, of 0.60–0.80 as good, and of 0.80–1.00 as excellent [21].

For sensitivity to change, the mean and median mSASSS and CASSSS status and progression scores were reported and the standardized response mean (SRM: the average of the progression scores divided by SD of the progression scores) was calculated. An SRM <0.5 was interpreted as small, 0.5–0.8 as moderate, and >0.8 as large [22]. In addition, the percentage of patients with maximal scores at baseline (ceiling effect) (and thus, not able to be identified with progression according to the scoring methods), and the percentage of patients with progression >0, with progression ≥ the SDC, and with definite progression during 4 years of follow-up were reported.

#### Truth

The truth aspect of the OMERACT filter focuses on the questions: “Is the measure truthful, does it measure what is intended? Is the result unbiased and relevant?” Information about the relevance of scoring the cervical facet joints was investigated by examining the absolute differences in status and progression scores of the CASSS compared to the mSASSS using the Wilcoxon signed-rank test. The CASSS and mSASSS had a common element of construct validity because they both included the anterior elements of the spine. The construct validity of incorporating assessment of the cervical facet joints in the mSASSS was evaluated by comparing the damage and progression of damage in the cervical facet joints to the vertebral bodies scored in the mSASSS.

Correlation between the mSASSS and the total facet joint score was investigated using Spearman’s correlation coefficient. In addition, the correlation between the mSASSS or the CASSS and spinal mobility measures (cervical rotation, occiput-to-wall distance, lateral spinal flexion, modified Schober test, and chest expansion), physical function (Bath AS functional index (BASFI)), and quality of life (AS quality of life (ASQoL) questionnaire) was investigated using Spearman’s correlation coefficients. Correlation of 0.00–0.20 was interpreted as very weak, 0.20–0.40 as weak, 0.40–0.60 as moderate, 0.60–0.80 as strong and 0.80–1.00 as very strong [21]. Data on cervical rotation were only available at 4 years in the GLAS cohort. [12] Therefore, correlation between scores and this measurement was investigated at 4 years instead of at baseline.

### Additional statistical analysis

Descriptive statistics were used; results were expressed as number of patients (%), mean ± SD or median (IQR) for categorical, normally distributed and non-normally distributed data, respectively. The chi-square or Fisher’s exact test, independent samples *t* test, and Mann-Whitney *U* test were used as appropriate to compare groups. A *p* value ≤0.05 was considered statistically significant. Statistical analysis was performed using IBM SPSS Statistics 22 (SPSS, Chicago, IL, USA).

## Results

### Study population

The baseline characteristics of the 98 patients with AS for whom spinal radiographs were available are described in Table 1. Patients had high disease activity at baseline, which reduced rapidly after start of TNF-α blocking therapy and stabilized during follow up (mean Bath ankylosing spondylitis disease activity index (BASDAI) was 3.0 ± 2.1, mean ankylosing spondylitis disease activity score (ASDAS) 2.0 ± 0.9, and median C-reactive protein (CRP) 3 (IQR 2–7) at 4 years). Baseline characteristics such as male gender, age, symptom duration, and human leukocyte antigen (HLA)-B27 status were comparable to other AS cohorts.

**Table 1:** Baseline characteristics of the AS study population

	All patients	Patients with available CASSS
	n = 98	n = 89
Male gender	74 (76)	66 (74)
Age (years)	41.8 ± 11.2	41.0 ± 11.1
Symptom duration (years)	16 (7–24)	16 (7–24)
Time since diagnosis (years)	7 (2–15)	7 (2–16)
HLA-B27+	82 (84)	74 (83)
BMI (kg/m^2^)	26.1 ± 3.5	25.4 ± 3.0
Current smoker	30 (37)	28 (38)
Total smoking duration (years)	12 (0–25)	12 (0–24)
History of IBD	9 (9)	9 (10)
History of uveitis	27 (28)	25 (28)
History of psoriasis	11 (11)	11 (12)
Peripheral arthritis	21 (21)	18 (20)
NSAID use	79 (85)	72 (85)
DMARD use	23 (23)	23 (26)
BASDAI (0–10)	5.9 ± 1.6	5.8 ± 1.7
ASDAS_CRP_	3.8 ± 0.8	3.8 ± 0.8
CRP (mg/L)	15 (7–25)	15 (7–25)
Occiput-to-wall distance (cm)	5.0 (0.0–11.1)	4.5 (0.0–10.0)
Lateral spinal flexion (cm)	7.9 (5.0–11.5)	8.1 (5.5–11.7)
Modified Schober test (cm)	3.0 (1.2–4.0)	4.5 (0.0–10.0)
Chest expansion (cm)	3.0 (2.0–4.0)	3.0 (2.0–4.0)
BASFI (0–10)	5.6 (3.7–7.1)	5.5 (3.6–7.1)
ASQoL (0–18)	10 (7–13)	9 (7–12)

Values are presented as number of patients (%), mean ± SD, or median (IQR). Total smoking duration: current and past smoking. Peripheral arthritis: presence of ≥1 swollen joints (range 0–44). *AS* ankylosing spondylitis, *CASSS* combined AS spine score, *HLA* human leukocyte antigen, *BMI* body mass index, *NSAID* non-steroidal anti-inflammatory drug, *DMARD* disease-modifying antirheumatic drug, *BASDAI* bath AS disease activity index, *ASDAS* AS disease activity score, *GDA* global disease activity, *CRP*, C-reactive protein, *BASFI* bath AS functional index, *ASQoL* AS quality of life questionnaire

### Feasibility

CASSS was assessed on the same cervical radiographs as used for the mSASSS and the assessment took only a few extra minutes. Total scores for the mSASSS were calculable in 94 (96%) patients and total scores for CASSS were calculable in 89 (91%) patients; >3 vertebral edges and ≥1 facet joint were not visible in 2 patients, >3 vertebral edges were not visible in 2 patients, and ≥1 facet joint was not visible in 5 patients. Facet joints at the levels of lower C6 to upper T1 and/or C6–C7 were not visible in these patients.

For further comparison, we used 89 patients with AS for whom radiographic data were available for both the mSASSS and CASSS in all analyses. The baseline characteristics of these patients are shown in Table 1. The nine patients without CASSS data were significantly older, had higher body mass index (BMI), worse spinal mobility, and worse quality of life.

### Discrimination

#### Reliability

**Fig. 1:**
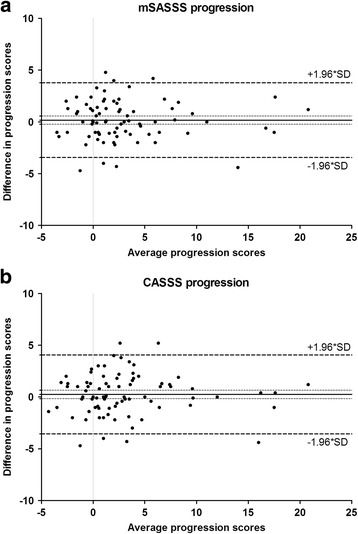
Bland and Altman plots show differences between readers in progression scores in assessed using the modified Stoke ankylosing spondylitis spine score (*mSASSS*) (**a**) and the combined ankylosing spondylitis spine score (*CASSS*) (**b**), plotted against the average scores of both readers and the 95% limits of agreement (+/− 1.96*SD)

Inter-observer reliability was excellent and was similar for the mSASSS and CASSS with ICCs >0.99 for status scores and 0.92 for progression scores. Bland and Altman plots revealed no systematic error in either scoring method (Fig. 1).

For mSASSS, Cohen’s kappa was 0.86 for the presence of definite damage and 0.87 for the development of definite damage, with percentages of absolute agreement of 93% for both definite damage and definite progression. For CASSS, the reliability and absolute agreement was even better; Cohen’s kappa was 0.98 for the presence of definite damage and 0.89 for the development of definite damage, with percentages of absolute agreement of 99% and 94%, respectively.

#### Sensitivity to change

At the group level, mean status scores at baseline were 19.3 ± 18.7 for the mSASSS and 21.8 ± 21.3 for the CASSS, showing large variation in radiographic damage according to both methods. Mean progression scores over 4 years of follow up were 2.7 ± 4.6 and 3.0 ± 4.8, respectively. The SDC using the mSASSS or the CASSS was smaller than the mean 4-year progression rate (Table 2). The SRM was 0.59 for the mSASSS and 0.63 for the CASSS (Table 2).

**Table 2:** Status and progression scores and proportion of patients with damage and progression according to the mSASSS and the composite CASSS

	mSASSS (range 0–72)	CASSS (range 0–87)
Status scores		
Observed range	0–72	0–87
Mean ± SD	19.3 ± 18.7	21.8 ± 21.3
Median (IQR)	11.3 (5.1–29.9)	13.5 (5.4–36.6)
Definite damage	51 (57)	54 (61)
Maximum score	3 (3)	1 (1)
Progression scores		
Observed range	−3.5–20.8	−4.3–20.8
Mean ± SD	2.7 ± 4.6	3.0 ± 4.8
Median (IQR)	1.2 (0.0–3.6)	1.6 (0.0–4.2)
SDC	1.8	1.9
SRM	0.59	0.63
Progression >0	64 (72)	64 (72)
Progression ≥ SDC	40 (45)	41 (46)
Definite progression	43 (48)	49 (55)

Values are presented as mean ± SD, median (IQR), or number of patients (%). *mSASSS* modified stoke ankylosing spondylitis spine score, *CASSS* combined ankylosing spondylitis spine score, *SDC* smallest detectable change, *SRM* standardized response mean

At the individual patient level, the CASSS resulted in more patients identified with definite damage at baseline (61% vs. 57%), fewer patients with the maximum score (1% vs. 3%), and more patients with definite progression during follow up (55% vs. 48%) (Table 2).

**Fig. 2:**
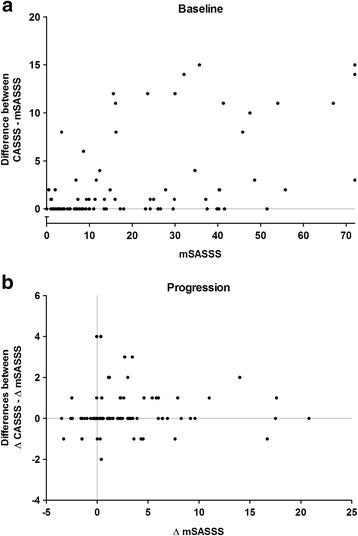
Differences in status and progression scores when comparing the composite score (combined ankylosing spondylitis spine score (*CASSS*)) (range 0–87) with modified Stoke ankylosing spondylitis spine score (*mSASSS*) (range 0–72). *X-axis* mSASSS status scores at baseline (**a**) and progression scores during 4 years of follow-up (**b**). *Y-axis*: differences between mSASSS and CASSS status and progression scores

### Truth

In comparison to the mSASSS, scoring radiographic damage using the CASSS resulted in 41 patients (46%) with higher baseline scores and 22 (25%) with higher progression scores (Fig. 2). Ten patients (11%) had lower progression scores as a result of scoring with unknown time sequence. Differences in scores were seen over the whole spectrum of the mSASSS, both in patients without damage and in patients with very advanced disease according to the mSASSS. No fan-out pattern was observed.

There was moderate correlation between the cervical facet joint score and the mSASSS (Spearman’s rho = 0.49). There was weak correlation between change in score for the cervical facet joints over 4 years and change in the mSASSS (Spearman’s rho = 0.16).

For radiographic damage assessed using either the mSASSS or the CASSS, there was strong correlation with the occiput-to-wall distance and with lateral spinal flexion; there was moderate correlation with the modified Schober test; and there was weak correlation with chest expansion and with the BASFI. There was no correlation between damage identified by either the mSASSS or the CASSS and the ASQoL (Table 3). Cervical rotation at 4 years was somewhat better correlated with the CASSS than with the mSASSS (Table 3).

**Table 3:** Correlation between radiographic damage assessed using the mSASSS and CASSS and spinal mobility, physical function, and quality of life at baseline

	mSASSS	CASSS
Cervical rotation^a^	−0.56**	−0.63**
Occiput-to-wall distance	0.64**	0.67**
Lateral spinal flexion	−0.62**	−0.61**
Modified Schober test	−0.44**	−0.45**
Chest expansion	−0.33**	−0.35**
BASFI	0.24*	0.26*
ASQoL	0.03	−0.01

Correlation is expressed as Spearman’s rho. ^a^Analysis performed with 4 years of data. *mSASSS* modified stoke ankylosing spondylitis spine score, *CASSS* combined ankylosing spondylitis spine score, *BASFI* Bath ankylosing spondylitis functional index, *ASQoL* ankylosing spondylitis quality of life questionnaire. *Statistically significant correlation *p* ≤ 0.05. **Statistically significant correlation *p* ≤ 0.01

Additional analysis using only the cervical mSASSS (range 0–36) and the cervical CASSS (range 0–51) showed that the cervical CASSS was more strongly correlated with cervical rotation (Spearman’s rho = 0.68) than the cervical mSASSS (Spearman’s rho = 0.59).

## Discussion

This is the first study investigating the additional value of cervical facet joints in the evaluation of spinal radiographic outcome in patients with AS, a slowly progressive disease in which both the anterior and posterior elements of the spine are affected [1, 7]. The composite scoring method CASSS, which combines the assessment of damage at the cervical facet joints (according to de Vlam) with the assessment of damage at the anterior corners of the cervical and lumbar vertebral bodies (the mSASSS), was compared with the original mSASSS using the three aspects of the OMERACT filter: feasibility, discrimination, and truth [17, 18].

The feasibility of the CASSS was very good; no extra radiographs were needed, it took only a few extra minutes to perform the CASSS, and the cervical facet joints were clearly visible in >90% of the patients. The lumbar facet joints were not included in the new scoring method because previous studies have shown that lumbar facet joints are more difficult to score than cervical facet joints. A study in 73 patients with psoriatic arthritis (PsA) showed that lumbar facet joints could be scored on lateral and anterior-posterior radiographs in 73–82% of the patients whereas cervical facet joints could be scored in 93–95% of the patients [15]. In the study of de Vlam et al., the inter-observer and intra-observer reliability was only poor to moderate for scoring individual lumbar facet joints on oblique radiographs (weighted kappa for the stages ranged between 0.19 and 0.52), but there was moderate to good reliability for scoring individual cervical facet joints on lateral projection radiographs (weighted kappa ranged between 0.55 and 0.66) [10].

As spinal radiographic progression in AS is a slow and very heterogeneous process, a reliable scoring method is needed with low measurement error and good discriminatory properties to detect changes over time. In our study the inter-observer reliability was excellent for both the mSASSS and the CASSS, with comparable SDCs (1.8 and 1.9, respectively). This implies that, although it involves scoring additional sites, the CASSS could be used without an increase in measurement error. The SDCs were smaller than the mean mSASSS and CASSS progression rates over 4 years and therefore “real” changes could be assessed during 4 years of follow up using either scoring method.

The most important benefit of the CASSS over the mSASSS is that the CASSS had better discriminatory properties and better construct validity than the mSASSS. The composite scoring method detected more patients with definite damage at baseline (61% vs. 57%). Seven percent of the patients with evidence of definite progression during 4 years of follow up according to the CASSS did not have progression according to the mSASSS. In addition, almost half of all patients had higher status scores and 25% had higher progression scores according to the CASSS. Differences in scores were found over the whole spectrum of the mSASSS. This confirms that not only the vertebral bodies but also the facet joints are frequently involved in AS, even if no damage or progression is observed or when patients have very advanced disease according to the original mSASSS [10–12].

There was weak correlation between disease progression in the facet joints and progression in the vertebral bodies. This suggests that the damage does not develop in the facet joints synchronically with the most typical AS-related damage, i.e., the development of syndesmophytes. It has been thought that damage in the facet joints is not specific to AS as these joints are also affected by degenerative changes [23]. The most characteristic degenerative changes in the facet joints are osteophyte formation (more horizontally oriented, >45°), joint space narrowing, sclerosis, and joint irregularity [20]. In patients with degenerative disc disease who are candidates for lumbar spine surgery, facet joint osteoarthritis was not observed in the absence of disc degeneration [24]. It is thought that degenerative changes begin in the disc, followed by changes in the facet joints [20, 24]. Therefore, the whole structure at each vertebral level was evaluated in the present study in order to distinguish between AS-specific changes and degenerative changes.

In a USA population based study from 1970, cervical osteoarthritis (not specifically facet joint osteoarthritis) was very rare before the age of 45 years (prevalence <2%). The prevalence of cervical osteoarthritis was 19% in adults aged 45–64 years, and in adults aged 65 years or older it was 56–57% [25]. The hallmark radiographically demonstrated characteristic of AS is ankylosis of the vertebral column. In our study partial or complete facet joint ankylosis was already evident in 19% of the patients aged <45 years. In patients aged 45–64 years, 34% had partial or complete facet joint ankylosis and 33% of patients aged 65 years or older had ankylosis. Incident partial or complete ankylosis was evident within 4 years of follow up in 14% of our patients with AS; half of these patients were younger than 45 years. This shows that AS-related cervical facet joint involvement begins at a relatively young age in comparison to osteoarthritis.

In our previous analysis we demonstrated that facet joint damage is associated with longer symptom duration, higher disease activity (ASDAS and CRP), worse spinal mobility (occiput-to-wall distance), and presence of extra-articular manifestations (inflammatory bowel disease (IBD), uveitis, and psoriasis) [12]. These findings, in combination with the strong correlation between CASSS and cervical rotation, occiput-to-wall distance, and lateral spinal flexion, as found in the present study, highlight the fact that damage in the cervical facet joints is common in AS, and is also evident at a younger age. Incorporating cervical facet joint scores is therefore of clinical relevance and improves the construct validity of the assessment of spinal radiographic damage in AS.

Previously, researchers in the field of PsA also developed a composite scoring method, the psoriatic arthritis spondylitis radiology index (PASRI). This scoring method combines the mSASSS of at the level of lower T12 to upper S1 and lower C2 to upper C6, the cervical facet joints (presence or absence of ankylosis), and the sacroiliac joints (SI) score (modified New York criteria) [13]. Although intra-observer and inter-observer reliability are excellent for the PASRI (range 0–72) in AS [26], it is very difficult to reliably score status and progression of disease at the SI joints in AS [27]. Besides this, the most important advantage of the CASSS over the PASRI is that the original mSASSS remains intact and, therefore, it is possible to obtain separate data on the mSASSS and the cervical facet joints. In this way, the original mSASSS scores in new studies can easily be compared with scores from former studies using only the mSASSS.

Our study was the first to investigate radiographic evidence of progression of disease at the cervical facet joints in patients with AS who had active disease and had started treatment with TNF-α inhibitors. The results may not be generalizable to all patients with axial spondyloarthritis (SpA). As potent biologic agents such as TNF-α inhibitors are available for the treatment of active disease in AS, it is unethical to withhold this treatment long-term in patients with active disease. Therefore, it was not possible to compare radiographic evidence of progression at the cervical facet joints in patients with and without TNF-α inhibitors in order to evaluate the effect of treatment. Further validation of the CASSS is needed in patients with axial SpA at different stages of disease duration, disease activity, and treatment strategies, and in long-term follow up. Data on radiographic evidence of progression at the vertebral bodies (mSASSS) show that at >4 years of follow up at least are needed to show radiographic evidence of diminishing progression during treatment with TNF-α inhibitors in this disease of overall slow progression [4, 28].

The assessment was performed with unknown time sequence to diminish possible reader bias due to knowledge of the applied therapy. However, it caused some negative progression scores (in 11 patients according to the CASSS and in 19 patients according to the mSASSS). Excessive bone formation in AS is an irreversible process, and therefore, negative progression is a result of measurement error. Reading radiographs in chronological time order is more sensitive to detect changes but less appropriate with respect to reader bias when the applied treatment regimens are known [29]. Future studies should determine the discrimination and truth aspects of the CASSS scored in chronological time order when radiographs from patients receiving conventional and biological treatment are randomized.

## Conclusions

The CASSS seems a promising scoring method to evaluate spinal radiographic outcome in AS. In our prospective observational cohort study we demonstrated that standardized scoring of the cervical facet joints in addition to the mSASSS is a feasible and reliable modification that provides better construct validity than the original mSASSS. Furthermore, the composite CASSS captures more patients with radiographic evidence of spinal disease progression, which is very valuable in this heterogeneous and overall slowly progressing disease.

## Key messages

- Incorporation of the cervical facet joints in the mSASSS is feasible, reliable and relevant for the assessment of spinal radiographic outcome in AS
- The combined AS spine score (CASSS) captured more patients with AS with damage and progression and correlated better with cervical rotation than mSASSS
- The original mSASSS and the cervical facet joint score of de Vlam can easily be extracted from the CASSS

## Abbreviations

AS: Ankylosing spondylitis
ASAS: Assessment of spondyloarthritis
ASDAS: Ankylosing spondylitis disease activity score
ASQoL: AS quality of life questionnaire
BASDAI: Bath ankylosing spondylitis disease activity index
BASFI: Bath ankylosing spondylitis functional index
BMI: Body mass index
C: Cervical
CASSS: combined ankylosing spondylitis spine score
CRP: C-reactive protein
DMARD: Disease-modifying antirheumatic drug
GDA: Global disease activity
GLAS: Groningen Leeuwarden ankylosing spondylitis
HLA: Human leukocyte antigen
ICC: Intra-class correlation coefficient
IQR: Interquartile range
L: lumbar
MCL: Medical Center Leeuwarden
mSASSS: modified Stoke ankylosing spondylitis spine score
NSAID: Non-steroidal anti-inflammatory drug
OMERACT: Outcome Measures in Rheumatology Clinical Trials
PsA: Psoriatic arthritis
RCT: Randomized controlled trial
SD: Standard deviation
SDC: Smallest detectable change
SI: sacroiliac
SpA: Spondyloarthritis
SRM: Standardized response mean
T: Thoracic
TNF-α: Tumor necrosis factor-alpha
UMCG: University Medical Center Groningen
